# Sigma E Regulators Control Hemolytic Activity and Virulence in a Shrimp Pathogenic *Vibrio harveyi*

**DOI:** 10.1371/journal.pone.0032523

**Published:** 2012-02-23

**Authors:** Pimonsri Rattanama, Janelle R. Thompson, Natthawan Kongkerd, Kanchana Srinitiwarawong, Varaporn Vuddhakul, John J. Mekalanos

**Affiliations:** 1 Department of Microbiology, Faculty of Science, Prince of Songkla University, Hat Yai, Songkhla, Thailand; 2 Department of Biomedical Science, Faculty of Medicine, Prince of Songkla University, Hat Yai, Songkhla, Thailand; 3 Department of Civil and Environmental Engineering, Massachusetts Institute of Technology, Cambridge, Massachusetts, United States of America; 4 Department of Microbiology and Molecular Genetics, Harvard Medical School, Boston, Massachusetts, United States of America; Baylor College of Medicine, United States of America

## Abstract

Members of the genus *Vibrio* are important marine and aquaculture pathogens. Hemolytic activity has been identified as a virulence factor in many pathogenic vibrios including *V. cholerae*, *V. parahaemolyticus*, *V. alginolyticus*, *V. harveyi* and *V. vulnificus*. We have used transposon mutagenesis to identify genes involved in the hemolytic activity of shrimp-pathogenic *V. harveyi* strain PSU3316. Out of 1,764 mutants screened, five mutants showed reduced hemolytic activity on sheep blood agar and exhibited virulence attenuation in shrimp (*Litopenaeus vannamei*). Mutants were identified by comparing transposon junction sequences to a draft of assembly of the PSU3316 genome. Surprisingly none of the disrupted open reading frames or gene neighborhoods contained genes annotated as hemolysins. The gene encoding RseB, a negative regulator of the sigma factor (σ^E^), was interrupted in 2 out of 5 transposon mutants, in addition, the transcription factor CytR, a threonine synthetase, and an efflux-associated cytoplasmic protein were also identified. Knockout mutations introduced into the rpoE operon at the *rseB* gene exhibited low hemolytic activity in sheep blood agar, and were 3-to 7-fold attenuated for colonization in shrimp. Comparison of whole cell extracted proteins in the *rseB* mutant (PSU4030) to the wild-type by 2-D gel electrophoresis revealed 6 differentially expressed proteins, including two down-regulated porins (OmpC-like and OmpN) and an upregulated protease (DegQ) which have been associated with σ^E^ in other organisms. Our study is the first report linking hemolytic activity to the σ^E^ regulators in pathogenic *Vibrio* species and suggests expression of this virulence-linked phenotype is governed by multiple regulatory pathways within the *V. harveyi*.

## Introduction

*Vibrio harveyi* is one of several closely-related species of *Vibrio* that cause disease in marine organisms [1]–[3]. Outbreaks of highly virulent *Vibrio* strains have caused major losses to shrimp farmers in Thailand and elsewhere [4]–[8]. The mechanisms of pathogenesis in these vibrios are not clearly understood and are likely mediated in part by strain-specific virulence factors. Hemolytic activity has been linked to virulence in many species of *Vibrio* where several different classes of hemolysins have been described. The *V. harveyi* hemolysin (VHH) is a member of the broadly distributed thermolabile hemolysin (TLH) family [9]–[12] and appears to be sufficient for hemolytic activity in some, but not all, strains of *V. harveyi* [12], [13]. We have previously determined that the *vhh* gene is present in all *V. harveyi* isolates from both healthy and diseased marine animals collected in Southern Thailand [14]. However, hemolytic activity on blood agar was variable in the *vhh* bearing isolates, suggesting that hemolytic activity is influenced by additional factors.

In order to better understand mediators of hemolytic activity and virulence in *V. harveyi* we have investigated genes that control hemolytic activity in the shrimp-virulent *V. harveyi* strain PSU3316. Results of an initial transposon screen led us to hypothesize that regulators of the cell envelope stress sigma factor RpoE control the elaboration of *V. harveyi* hemolytic activity and that this phenotype correlates with virulence in shrimp. Recent work in *Vibrio vulnificus* has revealed that the regulatory network of RpoE controls virulence in a mouse model [15]. To investigate whether the RpoE-operon regulatory proteins mediate the virulence of *V. harveyi* we have carried out targeted mutagenesis, in vitro and in vivo activity assays, and proteomic analysis to identify genes differentially expressed in wild-type and non-hemolytic mutants. Surprisingly, none of the differentially regulated proteins or gene products of loci inactivated in hemolysis-attenuated transposon mutants were similar to known hemolysins. These results suggest that hemolysis activity *per se* may be a phenotype that is influenced by a variety of different factors including the RpoE-operon mediated cell envelope stress response of *V. harveyi*.

## Materials and Methods

### Bacterial strains and plasmids

All bacterial strains and plasmids used in this study are listed in Table 1. *V. harveyi* PSU3316 was isolated in 2004 from the hemolymph of a diseased shrimp (*Penaeus monodon*) in Southern Thailand, and an early passage of this strain was archived in glycerol at −80°C. PSU3316 was identified as being most similar to type strains of *V. harveyi* by biochemical testing, genome sequencing, and phylogenetic analysis of the *gyrB* gene [16]. Although the systematics of the *V. harveyi*-group is subject to debate based on emerging genomic data we have chosen to retain the original designation pending official taxonomic revision of type strains. PSU3545 is a spontaneous streptomycin-resistant (Sm^R^) derivative of PSU3316. Confirmation of virulence of *V. harveyi* PSU3316 and PSU3545 was performed by determining the LD_50_ of each strain in shrimp (*P. monodon*) and through a competition assay involving co-infection of these strains and measurement of hepatopancreas colonization levels. All strains were maintained in 20% glycerol at −80°C.

**Table 1:** Bacterial strains and plasmids used in this study.

Strain			Plasmid		
Description		Reference	Description		Reference
*E. coli* DH5α/λ*pir*	λ*pir* φ80*dlacZ*ΔM15 Δ(*lacZYA-argF)U169, recA1, hsdR1, deoR, thi-1, supE44, gyrA96, relA1*	[21]	pSC189	*mariner*-based transposon delivery plasmid, NCBI accession no. AY115560	[18]
*E. coli* BW20767	RP4-2-Tc::Mu-1kan::Tn*7* integrant *leu-63*::IS*10, recA1, zbf-5, creB510, hsdR17, endA1, thi, uidA*(Δ*Mlu*I)::*pir* ^+^	[19]	pDTR801	R6K *ori mob*, *sacB*, *bla*; suicide vector; Cm^r^;Ap^r^	This study
*V. harveyi* PSU3316	Wild-type isolate from a diseased shrimp	This study	pJT064	pSC189 derivative; Ap^R^; Km^R^; Cm^R^	This study
*V. harveyi* PSU4512	PSU3316 derivative *rseB*::TnJT064	This study	pJT084	pDTR801 derivative; Cm^R^	This study
*V. harveyi* PSU3545	PSU3316 Sm^r^; spontaneous mutant	This study	pPR142	pJT084 with 1228 bp internal fragment harboring *rseA/B* from PSU3545 cloned into the *Xba*I site	This study
*V. harveyi* PSU4030	PSU3545 derivative *rseB*::pJT084	This study	pPR143	pJT084 with 516 bp internal fragment harboring *rseB* from PSU3545 cloned into the *Xba*I site	This study
*V. harveyi* PSU4031	PSU3545 derivative *rseB*::pJT084	This study			

### Media and growth conditions

*Vibrio* strains were grown at 30°C on Luria-Bertani medium (LB) or Tryptic Soy Broth (TSB) containing 1% or 1.5% NaCl. When required, chloramphenicol (Cm) (2 µg mL^−1^) or streptomycin (Sm) (200 µg mL^−1^) was added into broth or agar. *Escherichia coli* DH5α/λpir and BW20767/λpir used for construction of pJT064 and pJT084 were grown at 37°C in LB broth supplemented with chloramphenicol (20 µg mL^−1^). Unless otherwise indicated, all cultures were incubated for 16–18 h.

### Sequencing and draft assembly of the PSU3316 genome

Libraries suitable for sequencing using the Illumina Genome Analyzer (Illumina, Inc.) were generated using a modified version of the standard Illumina GA protocol. 5 µg of genomic DNA from strain PSU3316 was sheared using Adaptive Focused Acoustic technology (Covaris, Inc.) to generate fragments 100–300 bp in length. Fragments were blunt-ended, A-tailed and ligated with T nucleotide overhang Illumina forked paired end-sequencing adapters (Illumina, Inc.) containing custom bar-codes for multiplex sequencing. Libraries were then PCR amplified for 16 cycles after identifying the optimum number of cycles using qPCR, sequenced to a depth of ∼6× and assembled into contigs using CLC Genomics Workbench 4 (Aarhus, Denmark). Genome fragments were uploaded to the Rapid Annotations using Subsystems Technology (RAST) server for annotation [17]. Genome regions containing open reading frames for genes and proteins identified in this study have been deposited at NCBI with accessions XXX-YYY (*to be provided with final submission*).

### Transposon mutagenesis of *V. harveyi*

The *mariner*-based transposon pSC189 (Km^R^) [18] containing a kanamycin resistance gene was modified by insertion of a chloramphenicol marker into the *Pst*I restriction site to obtain pJT064. This plasmid carries a transposon fragment which was designated as TnJT064. pJT064 was transformed into competent *E. coli* BW20767 [19]. Conjugation of *E. coli* donor strain and *V. harveyi* PSU3316 was performed by mixing each strain at ratio of 1∶1 on LB plates supplemented with 1% NaCl and incubation at 30°C for 6 h followed by selection of *V. harveyi* mutants carrying TnJT064 insertions on *Vibrio*-selective Thiosulfate citrate bile salt-sucrose agar (TCBS) containing chloramphenicol (2 µg mL^−1^).

### Screening transposon insertion mutants for hemolytic activity and shrimp virulence

TnJT064 transposon insertion mutants were screened for hemolytic activity by spotting cultures on sheep blood agar (PML Microbiologicals, USA) and then scoring for the appearance of a lytic zone after incubation at 30°C for 48 h. Wild type *V. harveyi* PSU3316 and its streptomycin resistant derivative PSU3345 are β-hemolytic in this assay and any mutant that caused incomplete hemolysis (defined here as α-hemolysis) were subjected to a second screen for virulence in shrimp. Cells grown overnight in TSB were harvested by centrifugation, washed, and suspended in a sterile saline solution. Shrimp were challenged by intramuscular injection of a 100 µl of saline suspensions of approximately 2.4×10^6^ CFU (corresponding to four times LD_50_ for wild-type PSU3316), of either selected transposon mutants, PSU3316, PSU3545, or sterile saline (mock infection control). The challenge experiment was repeated twice, first with three shrimp per challenge followed by a second experiment with seven shrimp per challenge. Differences in mortality after 18 h in wild-type strains (PSU3316 and PSU3545) and mutant strains with attenuated hemolysis were evaluated using the Fisher's exact test. Relative risk (RR) of mortality compared with the wild type-strain was also determined for each mutant.

### Characterization of genes involved in hemolytic activity

The identity of genes disrupted in hemolysis- and virulence- attenuated mutants was determined by PCR amplification and sequencing of the transposon insertion junctions using primers targeting outward facing priming sites in the TnJT064 transposon (Mar2018 and MarEC6) and arbitrary primers ARB6/7 and ARB2 which bind throughout the genome [20]. Nucleotide sequences from transposon insertion junctions were compared to the PSU3316 genome by BLAST and identified based on annotation of the corresponding open reading frames disrupted by the transposon insertion.

### *rseBC* disruption

A spontaneous streptomycin resistant *V. harveyi* PSU3316 (designated as PSU3545) was used as the parental strain in the construction of two independent insertions in the *rseB* gene by homologous recombination-based gene disruption [21] which is expected to also inactivate the downstream gene *rseC*. PCR amplification of inserts for gene disruption was performed using specific primers designated in this study (Table 2). The PCR products were cloned into pJT084 (a derivative of suicide plasmid pDTR801 which carries the R6K origin of replication , the RP4 *mob* region, the *sacB* gene for sucrose-based counter selection from pCVD422 [22] modified by removal of the *bla* gene encoding ampicillin resistance by partial *PstI* digestion (NEB) and addition of a chloramphenicol acetyltransferase (*cat*) gene for chloramphenicol resistance), transformed into *E. coli* BW20767/λpir and conjugated with PSU3545. *V. harveyi* mutants containing gene disruptions were selected on TCBS containing chloramphenicol (2 µg mL^−1^) and streptomycin (200 µg mL^−1^).

**Table 2:** Nucleotide sequences of PCR primers for gene disruption mutagenesis.

Primers	Primer sequence 5′→3′	Product size (bp)
rseA-F1676 rseB-R2903	CATGTCTCTAGATAGAGCAAGACCAAGAGAGCCGTGCATGTCTCTAGACCACCCTACATTCCAGTTACTCG	1228
rseB-F2410 rseB-R2925	CATGTCTCTAGAATGCGACTCACGACAAAGACCAG CATGTCTCTAGATTGGATGGAAACCTTCAGGCGTC	516

### Quantification of hemolytic activity

Hemolytic activity expressed by *rseBC* mutant and wild-type strains on solid media was visualized using a standard sheep blood agar assay [23], [24]. Cell-associated hemolytic activity was quantified as the percentage of red blood cells lysed in liquid suspension assay. In brief, bacterial strains were grown 18 h at 30°C in LB agar containing 1.5% NaCl.

Bacterial cells and sheep erythrocytes were washed twice with sterile artificial seawater or Artificial SeaWater (Mariscience) prior to the assay. Bacterial cell suspensions were adjusted to 1.5×10^8^ CFU mL^−1^ by optical density at 600 nm and 190 µl of each strain was mixed with 10 µl of packed sheep erythrocytes in 96-well microtiter plate and incubated at 30°C for 5 h. Supernatants were obtained by centrifugation (4000× *g*; 10 min; 4°C) and the amount of hemoglobin released from the lysed erythrocytes was determined by spectrophotometer at 540 nm. Isotonic artificial seawater (9 ppt) was used as a non-hemolytic negative control and complete hemolysis was induced through osmotic shock with deionized water. The percentage of hemolysis was calculated using the following formula: % Hemolysis = 100×(OD_540_ of sample - OD_540_ of negative control)/(OD_540_ of positive control - OD_540_ of negative control). Differences in hemolytic activity in wild-type and mutant strains were evaluated using the student's t-test SPSS version 14 software (SPSS Inc., Chicago, Illinois).

### Growth and competition assays during shrimp infections

Growth rates of *V. harveyi rseBC* mutants (PSU4030 and PSU4031) were compared to the wild-type strain PSU3545. Strains were grown in LB broth containing 1.5% NaCl for 16 h at 30°C and growth was quantified by optical density at 600 nm every 60 min. Colony morphologies were observed on sheep blood agar. The competitive index (CI) of mutant strains was determined in vivo using juvenile *L. vannamei* shrimp (10 to 13 g with a length of 4 to 5 inches). Shrimp were obtained from a farm in Pattani province, Thailand, and were maintained in a 70 L glass tank containing artificial seawater (salinity 17 ppt) at a temperature of 29±1°C for at least 7 days before testing. For competition assays, cell suspensions of wild-type PSU3545 (Cm^S^ Sm^R^) and a mutant strain (Cm^R^ Sm^R^) were mixed at a ratio of 1∶1 in sterile saline and then approximately 1×10^6^ CFU were injected intramuscularly into each shrimp. Each competition was performed in duplicate using 7 shrimp per group. Approximately 18 h post infection, the hepatopancreas of each infected shrimp was removed, homogenized, and the proportion of *V. harveyi* parental and mutant strains determined by plating on TCBS supplemented with streptomycin. After colonies were recovered several hundred were picked on to TCBS supplemented with chloramphenicol and streptomycin to determine the ratio of PSU3545 (Cm^S^ Sm^R^) to either PSU4030 and PSU4031 (Cm^R^ Sm^R^).

The CI was calculated as follows: CI = (number of mutant CFU/number of wild type CFU isolated from hepatopancreas)/(number of mutant CFU/number of wild type CFU in injected cell suspension). A CI of less than 1 indicates the ability of the mutant strain to infect shrimp is lower than the wild type. Statistical analysis of results obtained from competition assays were evaluated by the Mann-Whitney U test [25], [26].

### Characterization of whole cell proteins from *rseBC* mutant

Protein extracts of *rseBC* mutant (PSU4030) and wild-type *V. harveyi* (PSU3545) were investigated to identify differentially expressed proteins that may mediate hemolytic activity. Strains were grown on sheep blood agar at 30°C for 24 h. Whole-cell protein extracts were prepared by suspending bacterial cells in 1 mL phosphate-buffered saline (PBS). Cells were washed three times with PBS. The cell pellets were then resuspended in lysis buffer (7 M Urea, 2 M Thiourea, 4% CHAPS, 2% Pharmalyte 4–7, 40 mM DTT) and incubated at 4°C for 6 h. The supernatant was collected by centrifugation at 14,000× *g* for 5 min at 4°C and was kept at −70°C until required.

2-D gel electrophoresis was performed according to the manufacturer's protocol (GE Healthcare). Briefly, tested samples were cleaned by 2D clean-Up Kits (GE Healthcare) and the concentration of protein was determined using PlusOne 2-D Quant Kit (GE Healthcare). The test sample was then applied to Immobiline Drystrip (IPG gel strip) and was transferred to IPGphor system (GE Healthcare). Isoelectric focusing (IEF) was conducted in the first dimension at 300 V for 0.30 h, 1000 V for 0.30 h, 5000 V for 1.20 h, and then 5000 V for 0.25 h. (total 6.5 kVh). After IEF, the IPG gel strips were equilibrated in sodium dodecyl sulfate (SDS) equilibration buffer (6 M Urea, 75 mM Tris-HCl (pH 8.8), 29.3% glycerol, 2% SDS, 0.002% bromophenol blue) containing 1% DTT for 15 min followed by a further 15-min incubation in the same buffer containing 2.5% iodoacetamide. The strips were transferred onto 12.5% Tris-glycine SDS polyacrylamide gel and subjected to SDS-polyacrylamide gel electrophoresis as the second dimension. Electrophoresis was performed with miniVE electrophoresis system (Amersham Bioscience AB, Sweden) with an initial constant current of 10 mA/gel for 15 min followed by 20 mA/gel. Proteins were visualized by staining with Coomassie Brilliant Blue R250, which is compatible with subsequent mass spectrometry-based protein identification. Gels were scanned by ImageScanner and the protein spots were analyzed using an ImageMaster 2D Platinum (Amersham Bioscience AB, Sweden). The experiment was performed in duplicate. Differentially expressed proteins from mutant and wild type were analyzed by nano liquid chromatography–electrospray ionisation tandem mass spectrometry (nano LC–ESI-MS/MS). Proteins were identified using MS/MS ion search of the Mascot search engine (Matrix Science, London, UK) and nonredundant protein databases (NCBInr; National Center for Biotechnology Information, Bethesda, MD, USA) with the following parameters; taxonomy: other proteobacteria; fixed modifications: cysteine carbamidomethylation; variable modifications: methionine oxidation, three missed cleavages allowed, peptide tolerance of 1.2 Da, and MS/MS tolerance of 0.6 Da. The identification of proteins was based on the Probability-Based MOWSE (molecular weight search) scores, whereby individual ion scores of greater than 53 indicates significant identities (*p*<0.05).

## Results

### Transposon mutagenesis of *V. harveyi* and screen for hemolysis and virulence

Using the *mariner*-based transposon mutagenesis, 1,764 *V. harveyi* PSU3316 mutants were generated and screened for a loss of hemolytic activity on sheep blood agar. Five mutants displayed low hemolytic activity (Table 3) and were subsequently screened for virulence in shrimp at a dose of four times the LD_50_ of the wild-type strain (2.4×10^6^ CFU shrimp^−1^). This dose of wild-type *V. harveyi* PSU3316 (or PSU3545, see below) consistently resulted in 100% shrimp mortality. Hemolysis attenuated mutants exhibited lower rates of mortality (Fisher's exact test *p*<0.05) where the relative risk of mortality of mutant strains was 2.5 to 5-fold less than wild-type. Other randomly chosen transposon mutants without disruption of the hemolysis phenotype were not attenuated for virulence (data not shown), indicating that the acquisition of the transposon per se is not associated with reduced shrimp virulence. Shrimp infected with *V. harveyi* exhibited characteristic disease symptoms including lethargy, and upon dissection, their hepatopancreas appeared discolored.

**Table 3:** Transposon mutants with reduced hemolytic activity on sheep blood agar.

Mutants	ORF Annotation in PSU3316 genome^a^	Homologous ORF in reference genome BAA-1116	*E* value^b^	Mortality in shrimp^c^^,^ d (#dead/total)
1. EG6	DNA-binding transcriptional regulator CytR	VIBHAR00726	0	1/3 and 2/7
2. EH12	Putative cytoplasmic protein, probably associated with Glutathione-regulated potassium-efflux	VIBHAR00067	1E-91	1/3 and 2/7
3. FH3	σ^E^ factor regulatoryprotein RseB	VIBHAR03540	0	1/3 and 1/7
4. GG7	σ^E^ factor regulatoryprotein RseB	VIBHAR03540	0	1/3 and 2/7
5. FD6	Threonine synthetase	VIBHAR00941	0	2/3 and 2/7

a Based on Rapid Annotation using Subsystem Technology (RAST) of de novo assembled genome fragments from PSU3316.

b *E* values determined using NCBI BLASTN using the full open reading frame of PSU3316.

c Control challenges with wild-type PSU3316 or transposant mutants with wild-type hemolysis activity exhibited mortality of 100% at the challenge dose.

d Hemolysis attenuated mutants exhibited variable mortality that was statistically different from wild-type as evaluated by Fisher's exact test (*p*<0.05).

**Figure 1:**
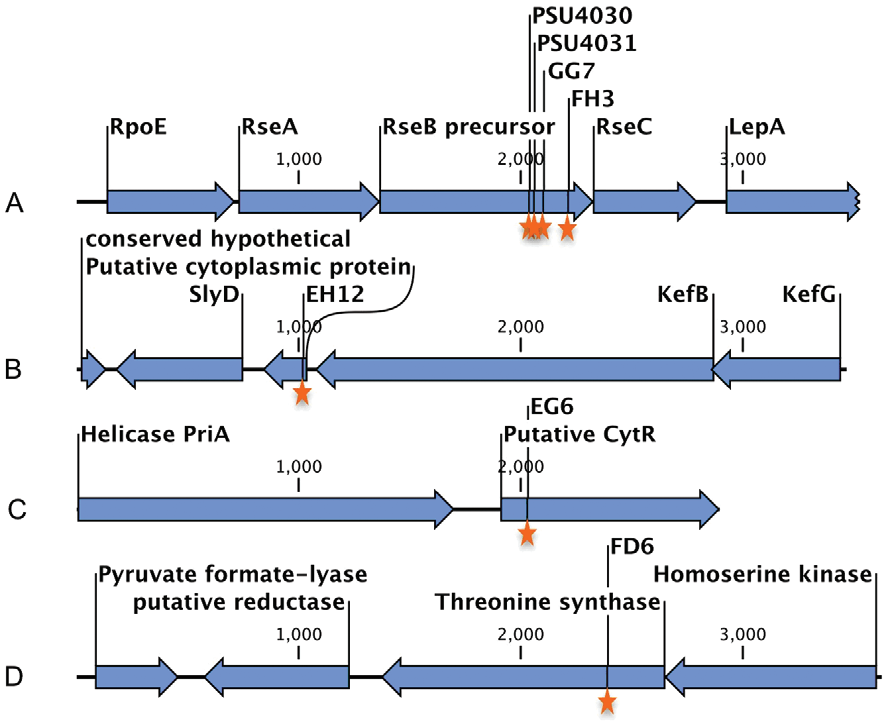
*V. harveyi* PSU3316 genome regions containing sites interrupted and the flanking open reading frames. Each region (A–D) was interrupted by transposon or targeted mutagenesis (orange stars). The corresponding full contigs from the draft genome assembly have been deposited at NCBI.

The DNA sequences corresponding to the junction of transposon insertions were determined for the attenuated *V. harveyi* mutants. Annotation of sequences adjacent to the transposon insertion sites revealed that 2 hemolysis-attenuated mutants (designated as FH3 and GG7) carried insertions that disrupted the gene encoding the σ^E^ factor negative regulatory protein (RseB) (Table 3 & Figure 1A). The other mutants carried insertions that interrupted genes encoding putative proteins annotated as a transcriptional repressor *cytR*, a threonine synthase, and a hypothetical protein in the *kefGB* operon associated with glutathione-regulated potassium-efflux (Table 3 & Figure 1B, C, D). These genes were also present in the genomes of closely-related strains (i.e. *V. harveyi* BAA-1116, *V. harveyi* HY01, and *V. parahaemolyticus* RIMD 2210633). Analysis of neighboring genes on contigs assembled from the *V. harveyi* PSU3316 genome and in the closely related *V. harveyi* genomes (BAA-1116 and HY01) did not reveal any known hemolysins that could be inactivated by polar effects of the transposon insertions characterized above.

### Involvement of rpoE regulators in the hemolytic activity of *V. harveyi*

**Figure 2:**
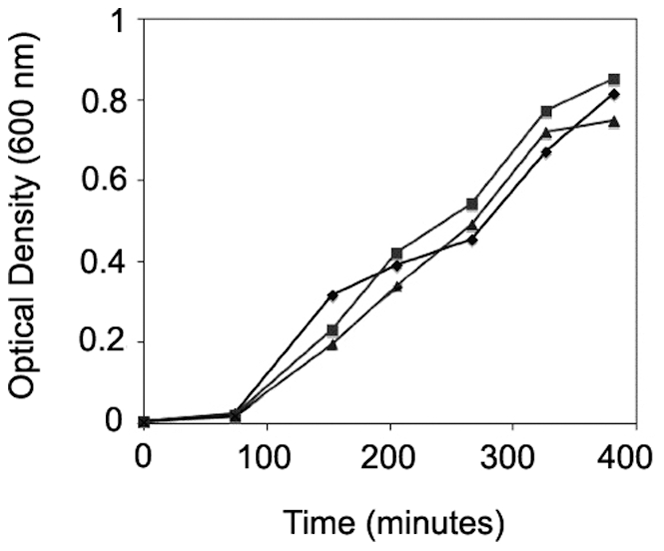
Growth of *V. harveyi* in LB broth with 1.5% NaCl, pH 7.5 at 30°C. **▪** Wild type (PSU3545), **⧫** RseBC^−^ (PSU4030), **▴** RseBC^−^ (PSU4031). No significant differences were observed in the growth of tested strains.

**Figure 3:**
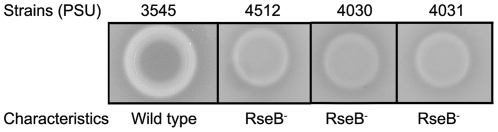
Hemolytic activity of wild type and mutants strains of *V. harveyi* on sheep blood agar. Images illustrate clear zone around the colony.

**Figure 4:**
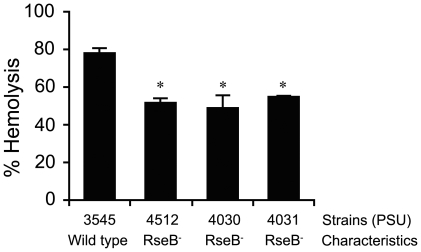
Percent hemolytic activity of *V. harveyi* wild type PSU3545 and *rseBC* mutants relative to controls. An asterisk indicates that a value is significantly different from the wild-type strain (PSU3545) (*p*<0.05). The error bars indicate standard errors of the means.

Since inactivation of the *rpoE* regulatory protein RseB was linked to reduced hemolytic activity, we hypothesized that this gene or the product of the downstream gene rseC may mediate hemolysin gene expression or activity. PSU3316 harbors a canonical *rpoE* operon structure comprised of four genes (*rpoE*-*rseA*-*rseB*-*rseC*) which are co-transcribed as a poly-cistronic operon in closely related model systems [27] (Figure 1A). Ideally, construction of in-frame mutations in all four of these genes would produce the appropriate a panel of mutations that might reveal the relationship between the *rpoE*-*rseA*-*rseB*-*rseC* operon and hemolytic activity in *V. harveyi* PSU3316. However, after repeated attempts to inactivate genes in this region and to create in-frame deletions using counter-selection, we were only able to construct two independent plasmid insertion mutations in *rseB* in *V. harveyi* PSU3545 using homologous recombination mediated by internal fragments of the *rseB* gene. Disruption of *rseB* is expected to also inactivate the downstream *rseC* gene. The two defined *rseBC* mutant strains (PSU4030 and PSU4031) displayed wild-type rates of growth in LB broth (Figure 2) and a defective hemolytic phenotype on sheep blood agar (Figure 3). Hemolytic activity of cell suspensions derived from these mutants strains grown on LB agar containing 1.5% NaCl was measured using a quantitative assay for hemoglobin release from sheep erythrocytes (see Materials and Methods). Compared to the parental strain PSU3545, hemolytic activity in the *V. harveyi rseBC* mutants (PSU4030 and PSU4031) was 20% lower but this reduction reached statistical significance (*p*<0.05) (Figure 4).

### *In vivo* competition assays

**Figure 5:**
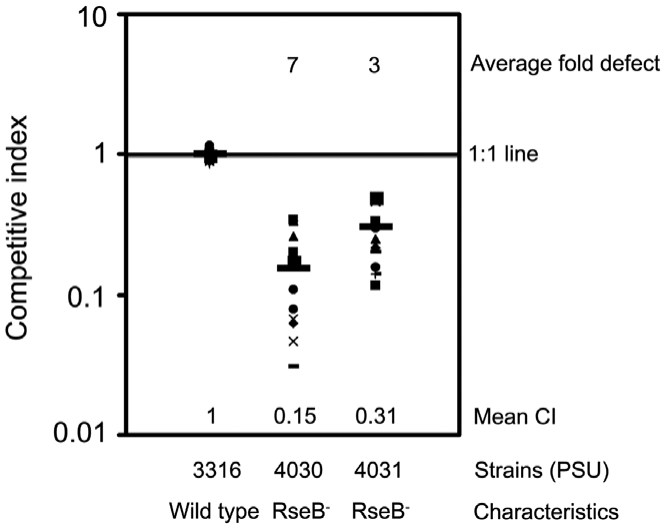
Competitive index of *V. harveyi rseBC* mutant strains relative to wild-type. For the competitive index assay shrimp were injected with mixture of wild type PSU3545 (Cm^S^) and *rseBC* mutant strains (PSU4030 or PSU4031) for approximately 1×10^6^ CFU shrimp^−1^. Values less than one indicate impaired colonization of the hepatopancreas after 18 h. An asterisk indicates that a value is significantly different from 1.0 (*p*<0.05).

The virulence of *V. harveyi rseBC* mutants (PSU4030 and PSU4031) was evaluated by quantification of shrimp colonization relative to wild-type *V. harveyi* (PSU3545). The competitive indexes (CI) for *rseBC* mutants PSU4030 and PSU4031 against the wild type were determined to be 0.15 and 0.31, respectively and both were significantly different from 1.0 (*p*<0.05) (Figure 5) indicating three to seven-fold reduction in the ability of either *rseBC* mutant to colonize shrimp relative to the wild-type strain.

### Comparative analysis and identification of proteins using 2D gel electrophoresis

**Figure 6:**
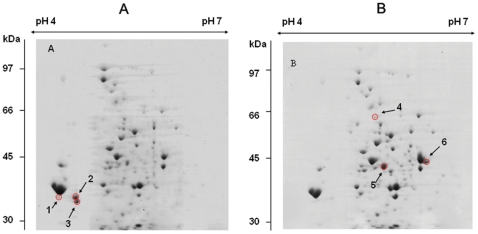
Two-dimensional gel electrophoresis of *V. harveyi* proteomes. (A) Wild type strain (PSU3545) and (B) *rseBC* mutant (PSU4030). Circled spots (numbers 1–6) represent differentially expressed proteins and were selected for mass spectrometry.

**Figure 7:**
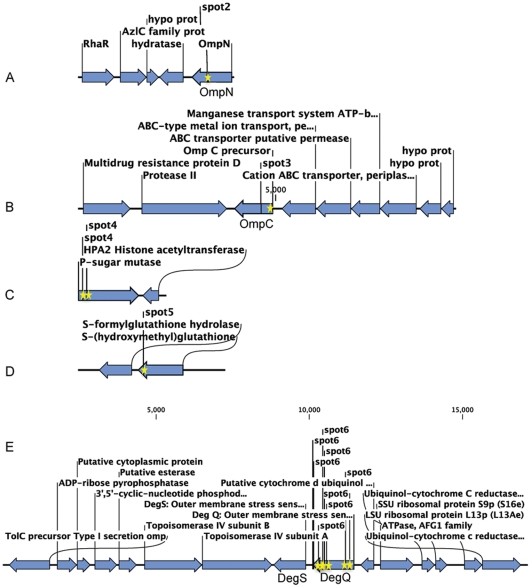
Contigs from the PSU3316 genome draft assembly bearing genes for differentially expressed proteins (A–E) in the *rseBC* mutant PSU4030 (spots 2 to 6, respectively). Peptide sequences from differentially expressed proteins were identified by mass spectrometry.

Two-dimensional protein gel electrophoresis revealed 6 proteins that were differentially expressed in the *rseBC* mutant (PSU4030) compared to the wild-type *V. harveyi* (PSU3545). These proteins were analyzed by nano LC-ESI-MS/MS and five proteins could be identified with significant identity (*p*<0.05) to proteins in the NCBInr database. In the non-hemolytic *V. harveyi rseBC* mutant, three proteins were significantly under-expressed (Figure 6-A, spots 1, 2, 3) corresponding to two outer membrane porins (OmpC-like and OmpN) and an unidentified protein (Figure 7-A, B; Table 4). Expression of three proteins was elevated in the *rseBC* mutant relative to the wild type (Figure 6-B, spots 4, 5, 6) corresponding to a phosphosugar mutase, S-(hydroxymethyl) glutathione dehydrogenase, and the protease (DegQ) (Figure 7 C, D, E & Table 4).

**Table 4:** Summary of MS/MS data for protein spots showing altered expression levels on 2-D gels for wild-type and *rseBC* mutant cell extracts.

Spot no.^a^	Predicted product in PSU3316^b^	Homolog in reference genome BAA-1116 (E value^c^)	Identified peptides	Ion score^d^	Theoretical p*I*/MW (kDa)
1	Unidentified	NA^e^	NA^e^	NA^e^	NA^e^
2	Outer membrane protein N, non-specific porin (ompN)	VIBHAR06284 (0.0)	IGYTYNGGDIQQANFVGK	109	4.53/37.80
3	Outer membrane protein C precursor (ompC)	VIBHAR06741 (3e-47)	LGYIGATHDQYGR	83	4.40/36.29
4	Phosphosugar mutase of unknown sugar	VIBHAR06273 (0.0)	GVVIGYDGRPDSKVAATPIVAFGVR	134	5.03/62.03
5	S-(hydroxymethyl)glutathione dehydrogenase	VIBHAR06925 (0.0)	SELPEIVNR	55	5.17/41.38
6	Outer membrane stress sensor protease DegQ, serine protease	VIBHAR00878 (0.0)	VTPAVVSIAVEGK GLGSGVIIDAK GAFVSQVVPDSAADK AIDTFSELR ITLGVIR GAELSNTTPSDKIQGVK GVLAINVQR TVYLVIR	538	5.88/48.03

a Spot no. corresponds to region of stained gel in Figure 6. Spots 1–3 were under-expressed and spots 4–6 were over expressed in the *rseBC* mutant relative to WT.

b Based on Rapid Annotation using Subsystem Technology (RAST) of de novo assembled genome fragments, or by homology to reference genome BAA-1116.

c *E* values determined using NCBI BLASTN using full open reading frame in PSU3316.

d Individual ion scores of greater than 53 indicates significant identities (*p*<0.05).

e Not available.

## Discussion

In the present study transposon and targeted insertion mutagenesis of *V. harveyi* PSU3316 revealed that disruption of regulatory elements in the rpoE operon modulate hemolytic activity and the ability of this pathogenic organism to colonize shrimp. The ability to lyse blood cells is an important virulence factor for *V. harveyi* [12] and other microbial pathogens [28] however modulation of hemolysis by the activity of RpoE operon regulatory proteins was previously unknown. In *E. coli* the σ^E^ operon contains the gene for the sigma(E) factor (*rpoE*) as well as the regulator elements *rseA*, *rseB* and *rseC* which are co-transcribed by a promoter upstream of the *rpoE* gene [29]. RseA and RseB are transmembrane and periplasmic negative regulatory proteins while RseC is a positive regulatory protein [30], [31]. Genome sequencing and draft assembly revealed an identical *rpoE*-*rseABC* operon structure in *V. harveyi* PSU3316 (Figure 1A). RpoE has been implicated in virulence as well as adaptive responses for survival in natural habitats [30], [32]–[35] and in Gram negative bacteria, stressors trigger accumulation of unfolded- or misfolded- proteins in the periplasm, and this in turn activates σ^E^ through RseA and RseB signal transduction [36], [37]. In *E. coli* the transcription factor encoded by *rpoE* (σ^E^) becomes more active when *rseB* is disrupted leading to increased transcription of σ^E^ dependent genes [27]. In *V. vulnificus rseB* mutants are attenuated for virulence in a mouse model through putative modulation of *rpoE* activity [15]. If regulation of the *rpoE* operon in *V. harveyi* is the same as in *E. coli* and as proposed for *V. vulnificus*, then the attenuated virulence phenotypes associated with the *V. harveyi rseBC* mutants may be due to changes in activity of RpoE due to relief of regulatory control by RseB or RseC. Studies in *E. coli* suggest deletion of the negative regulator *rseB* induces RpoE activity while deletion of the positive regulator *rseC* has a negligible effect [27], [30]. In *E. coli* the double mutant (*rseBC*) either has a net inducing effect on RpoE activity similar to a *rseB* deletion mutant (in the case of a transposon insertion in *rseB* with expected polar effects on *rseC*) [27] or revealed less induction of RpoE activity than the single *rseB* deletion mutant (in the case of a double deletion mutant in both *rseB* and *rseC*) suggesting competing effects of negative and positive regulation by RseB and RseC respectively on RpoE activity [30] In *V. harveyi*, if the activity of RseB is dominant to that of RseC then elevated RpoE activity in the *rseB* gene-disruption mutants would be responsible for alterations in protein expression that are negatively correlated with hemolysis and virulence. Alternatively, our transposon and plasmid insertions in *rseB* may have had a polar effect on expression of the downstream RpoE positive regulatory gene *rseC*. In the latter case, inactivation of either *rpoE* or *rseC* would be predicted to produce the same phenotype as such postulated polar *rseB* insertion mutations. Unfortunately we were not successful in making mutations in the *rpoE* region other than in *rseB* and it is possible that *rpoE* is an essential gene in *V. harveyi*. In addition, we were unable to select for in-frame deletions of *rseB* using sucrose- or streptomycin-based counter selection via the *sacB* or *rpsL* genes, respectively, due to the limited genetic tractability of this shrimp pathogenic *V. harveyi* strain. Additional genetic analysis (including the construction of double mutants in *rseB* together with *rseC* or *rpoE* and complementation with each gene) will be needed to resolve these two possible models for the phenotype associated with *rseB* insertion mutations.

To shed light on how disruption of the RpoE operon at the *rseB* gene controls virulence, we used proteome analysis to determine whether any protein level changes could be distinguished between non-hemolytic (*rseBC* mutant) and wild-type *V. harveyi* grown on sheep blood agar. We found that two outer membrane porins (OmpN- and OmpC-like) and an additional unidentified protein were decreased in the *rseBC* mutant relative to the wild-type (Table 4). This observation is consistent with down-regulation of OmpN during enhanced expression of σ^E^ in *E. coli* [38]. Several porins are established components of the RpoE regulon, controlling transport of solutes through the outer membrane and modulating the periplasmic environment [39]. Our data show that reduced porin expression and reduced hemolytic activity are both linked to disruption of the RpoE operon at the *rseB* gene in *V. harveyi* and characterization of these proteins and their potential involvement in RpoE-regulated virulence of *V. harveyi* will be a priority for future work in this system.

We also observed that three proteins were over-expressed in the *rseBC* mutant relative to the wild type. These proteins are a phosphosugar mutase, a S-(hydroxymethyl)glutathione dehydrogenase, and the DegQ protease which is homologous, and shares overlapping function with DegP [40], [41], a positively regulated member of the *rpoE* regulon in *E. coli* [42]. RpoE –directed regulation of DegQ in fish pathogenic *V. harveyi* has been suggested recently [41] and our data supports this model. It should be noted that differentially expressed RpoE, RseA, RseB, and RseC were not detected during proteomic analysis because the theoretical molecular weight of RpoE, RseA and RseC proteins were less than 30 kDa (21.7, 23.5 kDa, and 16.6 kDa respectively) and the theoretical pI of RseB was more than 7 (pI = 8.6) which were out of molecular weight and pI ranges investigated in this study.

Transposon mutagenesis, confirmed by targeted gene disruption, has allowed us to establish the first link between regulation of the RpoE-operon, hemolytic activity, and virulence in *V. harveyi*. This would not be possible in the closely-related and more genetically tractable *V. harveyi* strain BAA-1116 because it is weakly hemolytic and avirulent in the shrimp model used in this study. Thus, although limited by the availability of optimized genetic methods including in-frame deletion construction and complementation, analysis of non-laboratory adapted pathogens can provide insights into the regulation of virulence that may not be accessible in more highly characterized and optimized laboratory model systems. We have sequenced the genome of PSU3316 in anticipation of future optimized genetics.

In conclusion, we have demonstrated that disruption of RpoE operon at the *rseB* gene in a shrimp-pathogenic *V. harveyi* strain leads to reduction of hemolytic activity, shrimp colonization ability, and virulence. These changes are concomitant with changes in protein expression including decreased expression of two porins (OmpC-like and N) and increased expression of the stress-responsive periplasmic protease DegQ. If potential polar effects on *rseC* are negligible, as has been suggested in *E. coli* [27] our results suggest a model where enhanced activity of *rpoE* upon loss of *rseB* activity triggers periplasmic stress-responses that lead to diminished virulence by altering periplasmic homeostasis that controls some aspect of functional hemolytic activity. This study represents the first report of the role of the *rpoE* operon in the hemolytic activity of *V. harveyi* and paves the way for further work to determine the molecular mechanisms for hemolytic activity and virulence in this important group of marine pathogens.

## References

[1] Alvarez JD, Austin B, Alvarez AM, Reyes H (1998). *Vibrio harveyi*: a pathogen of penaeid shrimps and fish in Venezuela.. J Fish Dis.

[2] Pizzutto M, Hirst RG (1995). Classification of isolates of *Vibrio harveyi* virulent to *Penaeus monodon* larvae by protein profile analysis and M13 DNA fingerprinting.. Dis Aquat Org.

[3] Jiravanichpaisal P, Miyazaki T (1994). Histopathology, Biochemistry, and Pathogenicity of *Vibrio harveyi* Infecting Black Tiger Prawn *Penaeus monodon*.. J Aquat Anim Health.

[4] Leaño EM, Lavilla-Pitogo CR, Paner MG (1998). Bacterial flora in the hepatopancreas of pond-reared *Penaeus monodon* juveniles with luminous vibriosis.. Aquaculture.

[5] Kautsky N, Rönnbäck P, Tedengren M, Troell M (2000). Ecosystem perspectives on management of disease in shrimp pond farming.. Aquaculture.

[6] Liu PC, Lee KK, Chen SN (1996). Pathogenicity of different isolates of *Vibrio harveyi* in tiger prawn, *Penaeus monodon*.. Lett Appl Microbiol.

[7] Harris LJ, Owens L (1997). Guidelines for controlling luminous vibriosis in *Penaeus monodon*.. Austasia Aquacult.

[8] Liu PC, Lee KK, Yii KC, Kou GH, Chen SN (1996). Isolation of *Vibrio harveyi* from Diseased Kuruma Prawns *Penaeus japonicus*.. Curr Microbiol.

[9] Fiore AE, Michalski JM, Russell RG, Sear CL, Kaper JB (1997). Cloning, characterization, and chromosomal mapping of a phospholipase (lecithinase) produced by *Vibrio cholerae*.. Infect Immun.

[10] Kang JH, Lee JH, Park JH, Huh SH, Kong IS (1998). Cloning and identification of a phospholipase gene from *Vibrio mimicus*.. Biochim Biophys Acta.

[11] Shinoda S, Matsuoka H, Tsuchie T, Miyoshi S, Yamamoto S (1991). Purification and characterization of a lecithin-dependent haemolysin from *Escherichia coli* transformed by a *Vibrio parahaemolyticus* gene.. J Gen Microbiol.

[12] Zhang X-H, Meaden PG, Austin B (2001). Duplication of hemolysin genes in a virulent isolate of *Vibrio harveyi*.. Appl Environ Microbiol.

[13] Sun B, Zhang X-H, Tang X, Wang S, Zhong Y (2007). A single residue change in *Vibrio harveyi* hemolysin results in the loss of phospholipase and hemolytic activities and pathogenicity for turbot (*Scophthalmus maximus*).. J Bacteriol.

[14] Rattanama P, Srinitiwarawong K, Thompson JR, Pomwised R, Supamattaya K (2009). Shrimp pathogenicity, hemolysis, and the presence of hemolysin and TTSS genes in *Vibrio harveyi* isolated from Thailand.. Dis Aquat Org.

[15] Brown RN, Gulig PA (2009). Role of RseB, σ^E^, and DegP in virulence and phase variation of colony morphotype of *Vibrio vulnificus*.. Infect Immun.

[16] Thaithongnum S, Ratanama P, Weeradechapol K, Sukhoom A, Vuddhakul V (2006). Detection of *Vibrio harveyi* in shrimp postlarvae and hatchery tank water by the Most Probable Number technique with PCR.. Aquaculture.

[17] Aziz RK, Bartels D, Best AA, DeJongh M, Disz T (2008). The RAST Server: rapid annotations using subsystems technology.. BMC Genomics.

[18] Chiang SL, Rubin EJ (2002). Construction of a mariner-based transposon for epitope-tagging and genomic targeting.. Gene.

[19] Metcalf WW, Jiang W, Daniels LL, Kim SK, Haldimann A (1996). Conditionally replicative and conjugative plasmids carrying *lacZ* alpha for cloning, mutagenesis, and allele replacement in bacteria.. Plasmid.

[20] Cameron DE, Urbach JM, Mekalanos JJ (2008). A defined transposon mutant library and its use in identifying motility genes in *Vibrio cholerae*.. Proc Natl Acad Sci U S A.

[21] Miller VL, Mekalanos JJ (1988). A novel suicide vector and its use in construction of insertion mutations: osmoregulation of outer membrane proteins and virulence determinants in *Vibrio cholerae* requires *toxR*.. J Bacteriol.

[22] Donnenberg MS, Kaper JB (1991). Construction of an eae deletion mutant of enteropathogenic *Escherichia coli* by using a positive-selection suicide vector.. Infect Immun.

[23] Dasgupta N, Ashare A, Hunninghake GW, Yahr TL (2006). Transcriptional induction of the *Pseudomonas aeruginosa* type III secretion system by low Ca2+ and host cell contact proceeds through two distinct signaling pathways.. Infect Immun.

[24] Bag PK, Bhowmik P, Hajra TK, Ramamurthy T, Sarkar P (2008). Putative virulence traits and pathogenicity of *Vibrio cholerae* Non-O1, Non-O139 isolates from surface waters in Kolkata, India.. Appl Environ Microbiol.

[25] Hannan T, Mysorekar I, Chen S, Walker J, Jones J (2008). *LeuX* tRNA-dependent and-independent mechanisms of *Escherichia coli* pathogenesis in acute cystitis.. Mol Microbiol.

[26] Ilg K, Endt K, Misselwitz B, Stecher B, Aebi M (2009). O-antigen-negative *Salmonella enterica* serovar Typhimurium is attenuated in intestinal colonization but elicits colitis in streptomycin-treated mice.. Infect Immun.

[27] Missiakas D, Mayer MP, Lemaire M, Georgopoulos C, Raina S (1997). Modulation of the *Escherichia coli* sigmaE (RpoE) heat-shock transcription-factor activity by the RseA, RseB and RseC proteins.. Mol Microbiol.

[28] Honda T, Finkelstein RA (1979). Purification and characterization of a hemolysin produced by Vibrio cholerae biotype El Tor: another toxic substance produced by cholera vibrios.. Infect Immun.

[29] Erickson JW, Gross CA (1989). Identification of the sigma E subunit of *Escherichia coli* RNA polymerase: a second alternate sigma factor involved in high-temperature gene expression.. Genes Dev.

[30] De Las Penas A, Connolly L, Gross CA (1997). The σ^E^ -mediated response to extracytoplasmic stress in *Escherichia coli* is transduced by RseA and RseB, two negative regulators of σ^E^.. Mol Microbiol.

[31] Missiakas D, Raina S (1997). Protein folding in the bacterial periplasm.. J Bacteriol.

[32] Chi E, Bartlett DH (1995). An *rpoE*-like locus controls outer membrane protein synthesis and growth at cold temperatures and high pressures in the deep-sea bacterium *Photobacterium* sp. strain SS9.. Mol Microbiol.

[33] Hiratsu (1995). The *rpoE* gene of *Escherichia coli*, which encodes sigma E, is essential for bacterial growth at high temperature.. J Bacteriol.

[34] Gorham HC, McGowan SJ, Robson PRH, Hodgson DA (1996). Light-induced carotenogenesis in *Myxococcus xanthus*: light-dependent membrane sequestration of ECF sigma factor CarQ by anti-sigma factor CarR.. Mol Microbiol.

[35] Moreno MLG, Landgraf M (1998). Virulence factors and pathogenicity of *Vibrio vulnificus* strains isolated from seafood.. J Appl Microbiol.

[36] Mecsas J, Rouviere PE, Erickson JW, Donohue TJ, Gross CA (1993). The activity of σ^E^, an *Escherichia coli* heat-inducible -factor, is modulated by expression of outer membrane proteins.. Genes Dev.

[37] Rouviere PE, De Las Penas A, Mecsas J, Lu CZ, Rudd KE (1995). *rpoE*, the gene encoding the second heat-shock sigma factor, sigma E, in *Escherichia coli*.. EMBO J.

[38] Kabir MS, Yamashita D, Koyama S, Oshima T, Kurokawa K (2005). Cell lysis directed by σ^E^ in early stationary phase and effect of induction of the *rpoE* gene on global gene expression in *Escherichia coli*.. Microbiology.

[39] Nikaido H (1994). Porins and specific diffusion channels in bacterial outer membranes.. J Biol Chem.

[40] Kolmar H, Waller P, Sauer RT (1996). The DegP and DegQ periplasmic endoproteases of *Escherichia coli*: specificity for cleavage sites and substrate conformation.. J Bacteriol.

[41] Zhang W, Sun K, Cheng S, Sun L (2008). Characterization of DegQVh, a serine protease and a protective immunogen from a pathogenic *Vibrio harveyi* strain.. Appl Environ Microbiol.

[42] Ruiz N, Silhavy TJ (2005). Sensing external stress: watchdogs of the *Escherichia coli* cell envelope.. Curr Opin Microbiol.

