# Correction: Sigma E Regulators Control Hemolytic Activity and Virulence in a Shrimp Pathogenic *Vibrio harveyi*

**DOI:** 10.1371/annotation/de7188a8-1565-41fa-bc0c-418ea1bf8f44

**Published:** 2012-05-31

**Authors:** Pimonsri Rattanama, Janelle R. Thompson, Natthawan Kongkerd, Kanchana Srinitiwarawong, Varaporn Vuddhakul, John J. Mekalanos

The accession numbers were not provided in the manuscript text. Genome regions containing open reading frames for genes and proteins identified in this study have been deposited at NCBI with accessions JQ677126-JQ677134. 

